# Ixazomib, Oral Metronomic Cyclophosphamide, and Dexamethasone for First-Line Treatment of Multiple Myeloma: A Phase II Brown University Oncology Group Study

**DOI:** 10.1093/oncolo/oyad017

**Published:** 2023-03-03

**Authors:** Ari Pelcovits, Peter Barth, John L Reagan, Adam J Olszewski, Vallerie Rosati, Roxanne Wood, Ashlee Sturtevant, Eric S Winer

**Affiliations:** Department of Medicine, Alpert Medical School of Brown University, Providence, RI, USA; Division of Hematology-Oncology, Rhode Island Hospital, Providence, RI, USA; Department of Medicine, Alpert Medical School of Brown University, Providence, RI, USA; Division of Hematology-Oncology, Rhode Island Hospital, Providence, RI, USA; Department of Medicine, Alpert Medical School of Brown University, Providence, RI, USA; Division of Hematology-Oncology, Rhode Island Hospital, Providence, RI, USA; Department of Medicine, Alpert Medical School of Brown University, Providence, RI, USA; Division of Hematology-Oncology, Rhode Island Hospital, Providence, RI, USA; Division of Hematology-Oncology, Rhode Island Hospital, Providence, RI, USA; Division of Hematology-Oncology, Rhode Island Hospital, Providence, RI, USA; Division of Hematology-Oncology, Rhode Island Hospital, Providence, RI, USA; Division of Adult Leukemia, Dana-Farber Cancer Center, Boston, MA, USA

**Keywords:** multiple myeloma, ixazomib, cyclophosphamide, metronomic dosing

## Abstract

**Background:**

Newly diagnosed multiple myeloma patients have many available treatment options. While lenalidomide, bortezomib, and dexamethasone (RVD) is the preferred initial treatment for many patients, several other agents may provide similar efficacy with less toxicity and improved ease of administration.

**Methods:**

We evaluated the safety and efficacy of the all-oral regimen of ixazomib, cyclophosphamide, and dexamethasone with the use of metronomic cyclophosphamide dosing in the treatment of patients with newly diagnosed multiple myeloma.

**Results:**

The study was stopped prior to planned enrollment due to slow recruitment, with 12 patients available for final analysis. The overall response rate was 58.3% with 2 patients achieving a very good partial response (16.7%) and 5 patients achieving a partial response (41.7%). Median progression-free survival was 16 months, and median overall survival was 43 months. There were no episodes of grade 3 or greater peripheral neuropathy. Grade 3 or greater dermatologic toxicity was experienced in 50% of patients.

**Conclusion:**

Although limited enrollment prevented full efficacy evaluation, our data do not support further study of metronomic cyclophosphamide in combination with ixazomib and dexamethasone in the treatment of newly diagnosed multiple myeloma. The activity of this regimen in the relapsed/refractory setting requires further study (ClinicalTrials.gov Identifier: NCT02412228).

## Teaser

This phase IIBrown University Oncology Research Group trial investigated the effectiveness of an all-oral regimen of ixazomib, cyclophosphamide, and dexamethasone with the use of metronomic cyclophosphamide dosing in the treatment of patients with newly diagnosed multiple myeloma.

**Lessons Learned**

- Study enrollment for front-line therapy in multiple myeloma can be limited by the rapidly expanding landscape of available approved drugs in this setting.
- Toxicity rates for this regimen were higher than expected, with >40% of patients experiencing Grade 3 dermatologic adverse events, possibly impacting drug efficacy.
- The response rates and survival data with the combination of metronomic cyclophosphamide with ixazomib and dexamethasone for the treatment of multiple myeloma in the front-line setting do not support future study of this regimen in this space.

## Discussion

While lenalidomide, bortezomib, and dexamethasone (RVD), with the addition or substitution of daratumumab in recent years, is the preferred initial treatment for most patients with newly diagnosed multiple myeloma, there are several alternative options that may be of benefit to select patient subgroups. Lenalidomide is associated with significant risk of teratogenesis as well as diarrhea, cytopenias, hypokalemia, and an increased risk of venous and arterial thromboembolism.^1^ It is also largely eliminated by the kidneys; therefore, in patients presenting with renal insufficiency or failure, its use is relatively contraindicated. Bortezomib is often associated with the development of peripheral neuropathy and requires twice weekly clinic visits for injections, which can be difficult for elderly patients.

In this phase II Brown University Oncology Research Group trial, we investigated the effectiveness of an all-oral regimen of ixazomib, cyclophosphamide, and dexamethasone with the use of metronomic cyclophosphamide dosing in the treatment of patients with newly diagnosed multiple myeloma. Daily cyclophosphamide dosing, known as metronomic dosing, has been evaluated in several prior studies showing clinical response.^2^ Metronomic chemotherapy more broadly is thought to overcome tumor resistance and provide better therapeutic efficacy by targeting tumor vasculature through various endothelial–directed mechanisms.^3^ Ixazomib is the first oral proteosome inhibitor and has been shown to have less neurotoxicity than bortezomib, with ongoing evaluations of its efficacy in various combinations and time points in the treatment of multiple myeloma.^4^

**Figure 1.**
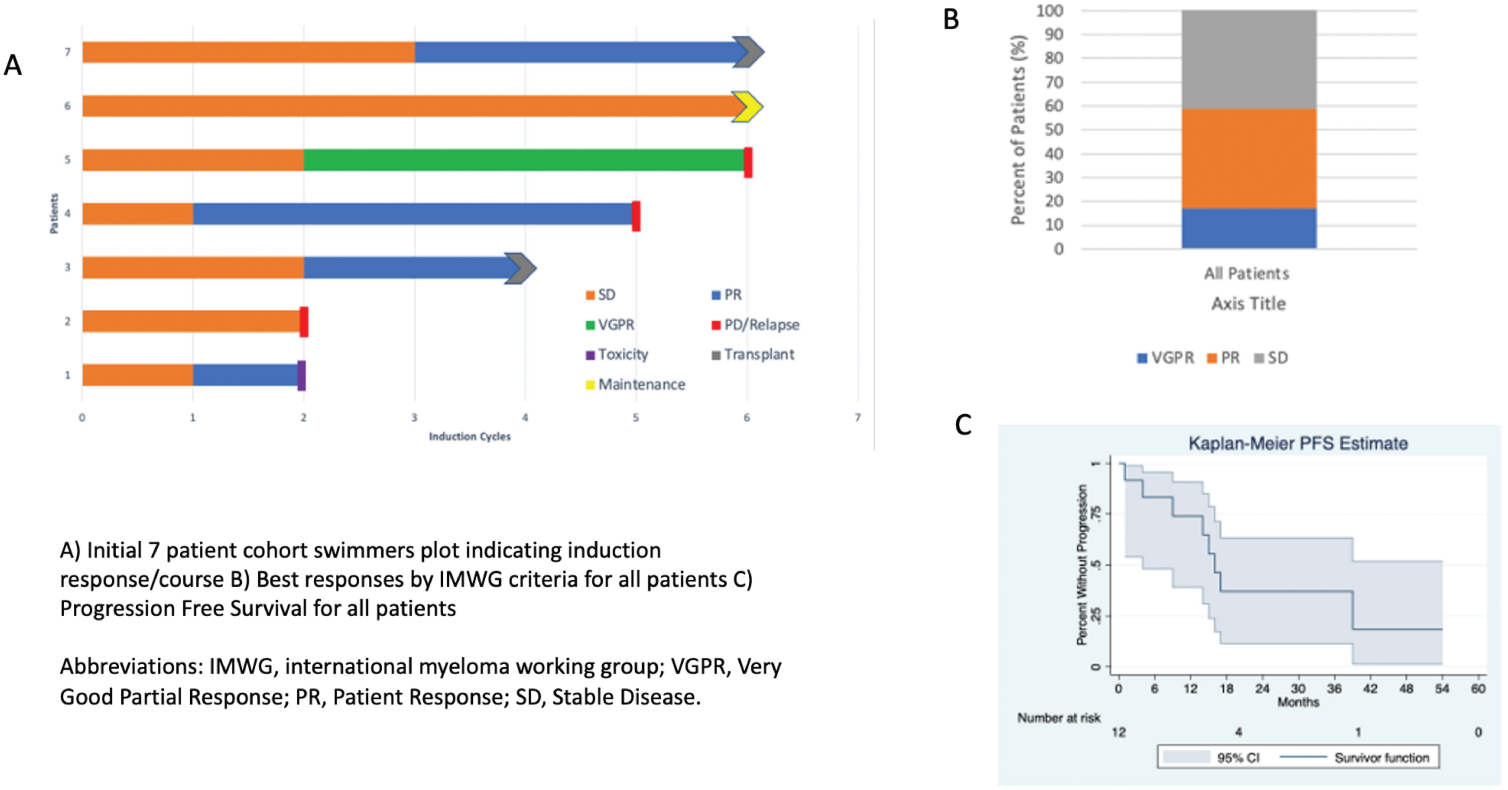
(**A**) Initial 7 patient cohort swimmers plot indicating induction response/course. (**B**) Best responses by IMWG criteria for all patients. (**C**) Progression-free survival for all patients. Abbreviations: IMWG, International Myeloma Working Group; VGPR, very good partial response; PR, patient response; SD, stable disease.

Simon’s 2 stage (MiniMax) design was used to evaluate response. Responses were determined using the 2016 IMWG response criteria and derived from the consensus guidelines for the uniform reporting of myeloma clinical trials.^5^ In the first stage, 5 or more responses in 7 enrolled patients were required to proceed (Fig. 1A). In the second stage, an additional 13 patients were to be enrolled for a total sample size of *N* = 20. If 14 out of 20 (70%) enrolled patients achieved a response, the null hypothesis would be rejected.

The study was terminated due to slow recruitment but with a total of 12 patients treated. Response rates and survival were below those seen in similar studies as well as below standard of care options, with only 16.7% of patients obtaining a very good partial response (VGPR) and a median progression-free survival (PFS) of 16 months (Fig. 1B, 1C). There are several possibilities for the low response rates including higher than expected rates of toxicity (42% of patients experienced a Grade 3 rash) and limited responses of the first 7 patients recruited (Fig. 1A). The investigators determined that these results would not warrant further study of this specific drug regimen.

**Table:** 

Trial Information	
Disease	Multiple myeloma
Stage of disease/treatment	Front-line therapy
Prior therapy	None
Type of study	Phase II
Primary endpoint	Response rate
Secondary endpoints	Toxicity, progression-free survival, overall survival
Investigator’s analysis	Level of activity did not meet planned endpoint

## Additional Details of Endpoints or Study Design

### Patients

From August 12, 2015 to March 29, 2019, a total of 18 patients underwent screening for the trial with 12 patients ultimately enrolled and treated. The trial was stopped prior to the 20 patients enrollment goal due to slow recruitment. Characteristics of the enrolled patients are listed in the Patient Characteristics section. Median age was 63.5 with a male predominance (83%). At the time of data cut-off 12 patients had completed a total of 62 cycles of treatment with the median number of cycles being 6 (range 2-9). Five of the 12 patients did not complete the initial 6 cycles of induction therapy (2 toxicity, 2 progression, and 1 early transplant after cycle 4). Only one patient proceeded to the maintenance phase of treatment and received 3 cycles of maintenance therapy before progression. Seven of the 12 patients ultimately went on to autologous stem cell transplantation.

### Study Treatment

In this phase II single-center trial all patients received the same induction treatment. Induction treatment consisted of 6 cycles of therapy. Cycle 1 consisted of ixazomib 4 mg and dexamethasone 20 mg each given once weekly on days 1, 8, and 15 with continuous cyclophosphamide at a dose of 50 mg per day. Cycle length was 28 days with continuation of cyclophosphamide during week 4 of each cycle. If treatment was tolerated in cycle 1 (see below) patients then received ixazomib 4 mg and dexamethasone 20 mg twice weekly (on days 1, 4, 8, 11, 15, and 18) during cycles 2-6 with no change in the continuous cyclophosphamide (50 mg/day). Cycle lengths remained 28 days for the remaining 5 cycles.

To proceed from once-weekly cycle 1 to twice-weekly cycles 2-6 dosing, patients could not have experienced grade ≥3 neutropenia or thrombocytopenia, febrile neutropenia, treatment related grade ≥3 non-hematology toxicity or treatment related grade ≥2 neuropathy. If any of the above were experienced, dose modifications were implemented which can be found in the Supplementary Appendix.

After the completion of 6 cycles of induction therapy, patients had the option of proceeding to autologous stem cell transplantation or maintenance therapy. Patients who went on to autologous transplant came off study and were followed per schedule evaluation. Patients not going on to autologous transplant continued with maintenance therapy which consisted of ixazomib at 4 mg days 1, 8, and 15 of a 28-day cycle for 1.5 years (to complete 2 years of protocol treatment) or until relapse.

### Objectives and Endpoints

The primary end point of this study was to evaluate the overall response rate to induction therapy (ORR). Response rates were evaluated using the International Myeloma Working Group Response Criteria. Secondary objectives included evaluation of toxicities, PFS, and overall survival (OS). Toxicities of interest included cytopenias, nausea, vomiting and diarrhea, rash, peripheral neuropathy, and posterior reversible encephalopathy. PFS was defined as the time from registration to the date of first documented progression or death. OS was defined as the time from registration to the date of death.

An ORR of ≥80% was judged as promising for further evaluation. Further evaluation would not be warranted if the ORR was <50%. Simon’s 2 stage (MiniMax) design was used. In the first stage, 5 or more responses in 7 enrolled patients were required to proceed. In the second stage, an additional 13 patients were to be enrolled for a total sample size of *N* = 20. If 14 out of 20 (70%) enrolled patients achieved a response, the null hypothesis would be rejected. This design yielded a type I error rate of 0.0417 and power of 81% when the true response rate is 80%. All patients must have received at least 2 cycles of treatment and have had a response post-cycle 1/pre-cycle 2 and post cycle 2/pre-cycle 3, at a minimum. If a patient did not have a confirmatory response at these two time points, additional time points would be needed until there is a confirmation.

**Table:** 

Drug Information	
Generic/working name	Ixazomib
Company name	Takeda
Drug type	Pro-apoptotic
Drug class	Proteasome inhibitor
Dose	4
Unit	mg
Route	PO
Schedule of administration	28 day cycles: weekly cycle 1, twice weekly (days 1, 4, 8, 11, and 15) cycles 2-6, and if not proceeding to transplant then days 1, 8, and 15 of 28 day cycles for 1.5 years maintenance
Generic/working name	Cyclophosphamide
Company name	Generic
Drug type	Cytotoxic
Drug class	Alkylating
Dose	50
Unit	mg
Route	PO
Schedule of administration	Daily during cycles 1-6
Generic/working name	Dexamethasone
Company name	Generic
Drug type	Immunosuppressive, anti-inflammatory
Drug class	Glucocorticoid
Dose	20
Unit	mg
Route	PO
Schedule of administration	28 day cycles: weekly cycle 1, twice weekly (days 1, 4, 8, 11, and 15) cycles 2-6

**Table:** 

Patient Characteristics	
Number of patients, male	10
Number of patients, female	2
Age: median (range)	63.5 (42-70) years
Number of prior systemic therapies	0
Performance Status: Eastern Cooperative Oncology Group	0: 8
	1: 4
	2: 0
	3: 0
	4: 0
Cancer types or histologic subtypes	IgG, 4; IgA, 4; serum-free light chain only, 3; other, 1

**Table:** 

Primary Assessment Method	
Number of patients screened	18
Number of patients enrolled	12
Number of patients evaluable for toxicity	12
Number of patients evaluated for efficacy	12
Evaluation method	International Myeloma Working Group (IMWG) response criteria
Response assessment (best response)	VGPR: 2 (16.7%)
	PR: 5 (41.7%)
	SD: 5 (41.7%)
	PD: 0 (0%)
Duration assessments (median)	PFS: 16 months (CI: 4-NR)
	OS: 43 months (CI: 28-NR)

## Outcome Notes

### Primary Endpoint

**Figure 2.**
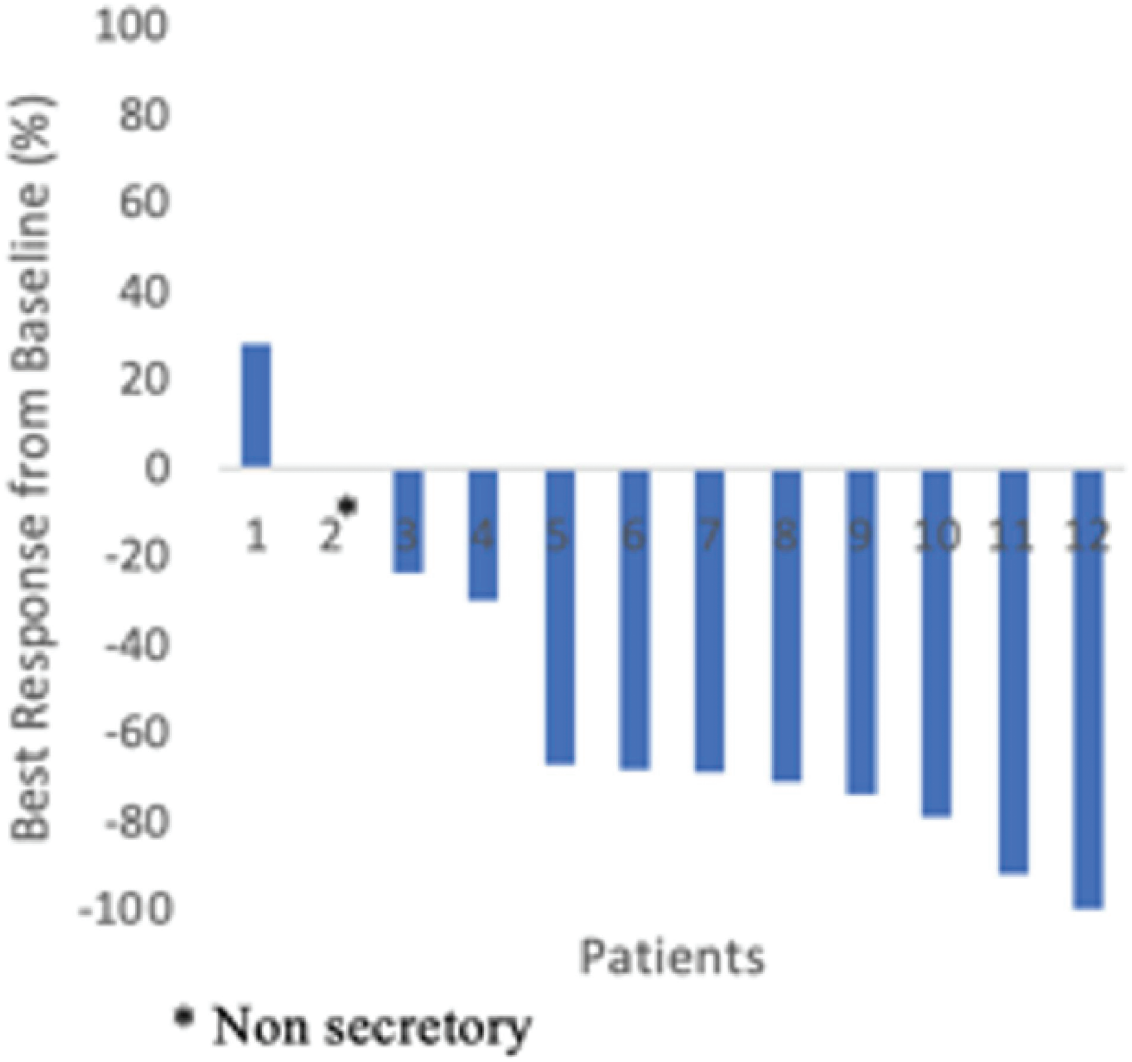
Best response by M protein/free light chain.

The median duration of follow up for the entire study group was 30 months (range 14-66) The ORR was 58.3% with 2 patients achieving a VGPR (16.7%) and 5 patients achieving a partial response (41.7%) (Fig. 1B). Waterfall plot below depicts the best response of all patients on the trial (Fig. 2).

Per trial protocol, 7 patients were initially enrolled and evaluated for response. Amongst these 7 patients 5 responses were seen. Four patients had a best response of PR and 1 patient had a VGPR. Only 5 more patients were recruited prior to closure of the study for a total of 12 patients for evaluation of efficacy. Among these first 7 patients 3 patients ultimately had progressive disease by the end of initial induction therapy, including progression in 2 patients who initially had a response (1 PR and 1 VGPR). One patient discontinued treatment due to toxicity, a grade 3 maculopapular rash felt to be secondary to Ixazomib. By the end of induction only 2 out of the first 7 patients had completed induction therapy and continued to have a response but neither of these patients achieved a VGPR. A swimmer’s plot of these first 7 patients and their response course is depicted in Fig. 1A.

### Secondary Endpoints

**Figure 3.**
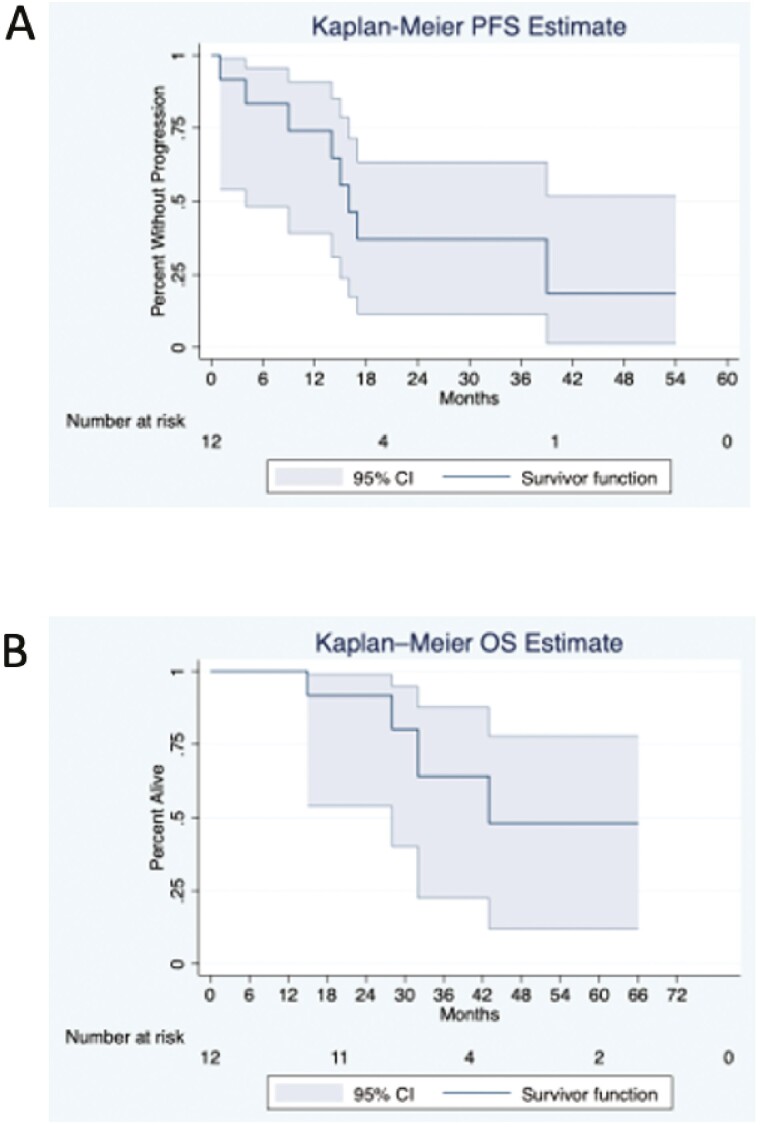
Progression-free survival (PFS) and overall survival (OS) estimates. (**A**) PFS estimates for all patients. (**B**) OS estimates for all patients.

Secondary objectives included evaluation of PFS and OS. Median PFS was 16 months (95% CI 4 to not reached) and Median OS was 43 months (95% CI 28 to not reached; Fig. 3).

### Safety and Toxicity

The safety profile of toxicities of interest is listed in Table 1, with a summary of all adverse event listed in Table 2. A complete list of toxicities can be found in the Supplementary Appendix. Two patients (16.7%) experienced grade 3 Adverse Events leading to discontinuation of study drugs (both maculopapular rash). Six patients required dose adjustment, with the most common reason being grade 3 maculopapular rash (4 patients), and 1 patient with grade 3 neutropenia, and 1 patient with grade 3 urticaria. There was one case of a serious adverse event in a patient with grade 3 enterocolitis.

**Table 1.** Toxicities.

Adverse effect	Total patients *N* = 12	
	Any grade	≥Grade 3
Anemia	8 (67%)	2 (17%)
Thrombocytopenia	9 (75%)	1 (8%)
Neutropenia	4 (33%)	3 (25%)
Lymphopenia	10 (83%)	5 (42%)
Nausea	5 (42%)	0 (0%)
Vomiting	1 (8%)	0 (0%)
Diarrhea	4 (33%)	1 (8%)
Rash	10 (83)	6 (50%)
Neuropathy	7 (58)	0 (0%)

**Table 2.** Adverse events.

Adverse event	*n* (range) or *n* (%)
Median treatment cycles, *n* (range)	6 (2-9)
Any adverse event	12 (100%)
Any Serious Adverse Events	1 (8%)
Any Grade ≥3 adverse event	10 (83%)
Adverse event resulting in dose reduction of any drug	6 (50%)
Adverse even resulting in discontinuation of any agent	2 (17%)
Adverse event resulting in discontinuation of the study regimen	2 (17%)
Death during the treatment period	0 (0%)

## Assessment, Analysis, and Discussion

**Table:** 

Completion	Study completed
Investigator’s assessment	Level of activity did not meet planned endpoint

In this phase II trial, we found that response rates and survival with oral metronomic cyclophosphamide, ixazomib, and dexamethasone for patients with newly diagnosed multiple myeloma were below those seen in other studies using similar drugs. In our study only 16.7% of patients obtained a VGPR and 58.3% of patients a PR or better (Fig. 1B, 1C). In a similar study conducted by Dimopoulos et al. using a weekly cyclophosphamide dosing scheme (in addition to ixazomib and dexamethasone) in transplant ineligible patients, 25% of patients obtained a response of CR or VGPR and 73% of patients obtained a PR or better.^6^ The median PFS was 23.5 months in that study when compared with 16 months in our study. In a randomized phase III trial comparing ixazomib, lenalidomide, and dexamethasone (ixazomib-Rd) to lenalidomide and dexamethasone alone (TOURMALINE MM2), median PFS for the ixazomib-Rd cohort was 35.3 compared to 21.8 months in the Rd group, and while this improvement in PFS did not meet statistical significance these are both longer than the PFS of 16 months seen in our trial.^7^ Additionally, 63% of patients in the ixazomib-Rd arm obtained a VGPR or better compared to the 16.7% of patients in our trial.

Response rates and survival are also superior in other standard of care options for front-line treatment for multiple myeloma. In patients who received RVD in the SWOG S0777 trial 27.8% of patients and 81.5% of patients achieved a VGPR or PR or better, respectively.^8^ Median PFS was 43 months in patients receiving RVD in SWOG S0777, and 36 months in the RVD alone arm of the IFM 2009 study (as compared to 50 months in patients who received RVD and autologous stem cell transplantation).^8,9^ Median OS was 43 months in our study, also shorter than median OS in the aforementioned studies (Fig. 3B). Median OS was 75 months in patients receiving RVD in SWOG S0777 (and 64 months in patients receiving lenalidomide and dexamethasone alone) and has yet to be reached for either arm in the IFM 2009 study with an 8-year OS rate of 60.2% and 62.2% in RVD alone and transplant groups, respectively.^8,10^

There are several possible reasons for the low response rate and worse survival seen in our study. Metronomic cyclophosphamide may be inferior to standard weekly cyclophosphamide dosing, especially in the newly diagnosed setting. Metronomic cyclophosphamide has been evaluated in several studies; however, its efficacy has largely been seen in the relapsed/refractory setting.^11,12^ The benefits of weekly cyclophosphamide however, in combination with a proteasome inhibitor (bortezomib) and dexamethasone (CyBorD), in the front-line setting have been demonstrated in several phase II studies, with long-term follow-up from one of these studies showing a median PFS of 40 months.^13,14^ In the previously referenced study by Dimopoulos et al, cyclophosphamide was delivered weekly and a longer PFS and higher response rate were seen.^6^ As our study was in effect evaluating two unique drug applications simultaneously (metronomic cyclophosphamide and the use of ixazomib in the front-line setting) it is not possible to ascertain which component may have contributed to reduced response.

Several other factors may have also limited the efficacy evaluation of our study. Enrollment to the study was limited by several factors and we did not reach our goal of 20 patients for full efficacy evaluation. Study enrollment was started in August 2015 and between initial protocol development and first cohort enrollment several studies have been published showing the efficacy of alternative treatment options. These include phase I/II data of carfilzomib-lenalidomide–dexamethasone (KRd) in 2015, the formal the publication of the SWOG S077 data in 2017, and the addition or substitution of daratumumab to the backbone of RVD as early as 2019.^8,15,16^ This may have led to patients choosing one of these alternative treatment options as opposed to enrolling on this study. Another factor likely limiting enrollment was the response profile of the initial 7 patients. While the prespecified goal of 5 responses was met allowing for progression to the second phase of the trial, these responses were not sustained in several patients and none of the first 7 patients ultimately completed induction in VGPR (Fig. 2). The limited responses of these first 7 patients may have deterred enrollment of future patients, especially with the approval of the new medications referenced above. We also saw higher rates of grade 3 dermatologic toxicity than has been seen in other studies, with 4 patients (42%) experiencing grade 3 maculopapular rash and 1 patient experiencing grade 3 urticaria, when compared with only 5% of patients experiencing grade 3 rash in the TOURMALINE-MM1 study group.^4^ Given the small sample size of our study we recommend interpreting these results with caution but this led to significant dose modifications that may have impacted outcomes.

While our sample size was limited our data do not suggest benefit of further study of this specific drug regimen in the first-line setting except in unique patient populations that may not be able to tolerate alternative treatment options. Given the larger patient size in the Dimopoulos et al. study (of all oral weekly cyclophosphamide, ixazomib, and dexamethasone) and the improved response rates it would suggest that an all-oral therapy with weekly cyclophosphamide as opposed to metronomic dosing could be considered.^6^

## Data Availability

The data underlying this article will be shared on reasonable request to the corresponding author.
